# Mid-Upper Arm Circumference Based Nutrition Programming: Evidence for a New Approach in Regions with High Burden of Acute Malnutrition

**DOI:** 10.1371/journal.pone.0049320

**Published:** 2012-11-26

**Authors:** Sylvie Goossens, Yodit Bekele, Oliver Yun, Géza Harczi, Marie Ouannes, Susan Shepherd

**Affiliations:** 1 Médecins Sans Frontières, Paris, France; 2 Médecins Sans Frontières, New York, New York, United States of America; Indiana University, United States of America

## Abstract

**Background:**

In therapeutic feeding programs (TFP), mid-upper arm circumference (MUAC) shows advantages over weight-for-height Z score (WHZ) and is recommended by the World Health Organization (WHO) as an independent criterion for screening children 6–59 months old. Here we report outcomes and treatment response from a TFP using MUAC ≤118 mm or oedema as sole admission criteria for severe acute malnutrition (SAM).

**Methods:**

Patient data from September 2007 to March 2009 for children admitted by MUAC ≤118 mm or oedema to a Médecins Sans Frontières (MSF) TFP in Burkina Faso were retrospectively analyzed. Analysis included anthropometric measurements at admission and discharge, program outcomes and treatment response.

**Results:**

Of 24,792 patient outcomes analyzed, nearly half (48.8%; n = 12,090) were admitted with MUAC 116–118 mm. Most patients (88.7%; n = 21,983) were 6–24 months old. At admission, 52.7% (n = 5,041) of those with MUAC 116–118 mm had a WHZ <−3 SD. At discharge, 89.1% (n = 22,094) recovered (15% weight gain or oedema resolution), 7.9% (n = 1,961) defaulted, 1.5% (n = 384) failed to respond to treatment, and 1.0% (n = 260) died. Average weight gain was 5.4 g/kg/day, and average MUAC gain was 0.42 mm/day. Patients with MUAC ≤114 mm at admission had higher average daily weight and MUAC gains at discharge compared to those admitted with MUAC 116–118 mm, but those in the latter category required longer lengths of stay to achieve recovery (*P*<0.001).

**Conclusion:**

This analysis suggests that MUAC ≤118 mm as TFP admission criterion is a useful alternative to WHZ. Regarding treatment response, rates of weight and MUAC gain were acceptable. Applying 15% weight gain as discharge criterion resulted in longer lengths of stay for less malnourished children. Since MUAC gain parallels weight gain, it may be feasible to use MUAC as both an admission and discharge criterion.

## Introduction

High prevalence of undernutrition in children less than 5 years old results in substantial levels of mortality and overall disease burden in low- and middle-income countries. Severe acute malnutrition (SAM), defined as severe wasting and/or nutritional oedema, is the form of undernutrition associated with the highest mortality risk [1]. Over the past decade great progress has been made in the treatment of SAM through community-based management including ready-to-use therapeutic food (RUTF), which has proven effective in supporting rapid weight gain and nutritional recovery [2], [3]. This is now the strategy recommended by the WHO, UNICEF, World Food Program, and UN Standing Committee on Nutrition [4]. However, the anthropometric criteria that define non-oedematous SAM (eg. severe wasting), and thus eligibility for community TFP, remain cumbersome, because it combines two independent forms of anthropometry: WHZ <−3 and MUAC <115 mm. The combination of 2 measurements unnecessarily complicates the admission process, and it does not appear that combining these two admission criteria improves the identification of children at highest risk of death, compared to using MUAC alone [5].

To implement TFPs in settings where resources and trained health professionals are scarce, simple diagnostic tools are needed. MUAC is a rapid method of assessing nutritional status, without requiring extensive training, supervision, or materials. With simple color-coding and on-the-spot interpretation, MUAC is relatively easy to use and simple to understand for both community health workers and children's caretakers. Errors of measurement associated with MUAC are no more frequent than with either weight or height. Studies have shown that even for minimally trained health workers, intra- and inter-observer reliability of MUAC measurements are at least as good if not superior to other anthropometric indices [6], [7]. In addition to reliability and simplicity, MUAC has demonstrated superior sensitivity to risk of death [8], [9], and can offer considerable cost advantages; MUAC tapes are less expensive than height boards.

Although MUAC does increase with age and height [10], [11], correcting arm circumference for either of these parameters appears to offer no advantage for predicting risk of death [12], [13]. For this reason, TFPs generally use one MUAC admission threshold for children 6–59 m, without adjustment for age [14]. MUAC-based programs will therefore exhibit a selection bias for young children, but this may be beneficial as those are also the most vulnerable to illness and at higher risk of death. Because of its multiple advantages, MUAC can be easily utilized in community-based TFPs, including emergencies, where the objective is to cover as large a number of severely malnourished children as possible, in situations where resources and close supervision may be limited.

In northern Burkina Faso, where food insecurity is a chronic concern, Médecins Sans Frontières (MSF) in collaboration with the Ministry of Health, implemented a TFP in 2007. Some focus group discussions, held by MSF staff with the caretakers of children admitted in TFP, have shown that mothers often do not link the wasting in children to health problems, and are unlikely to spontaneously seek medical help when their children lose weight, which shows the importance of including active case-finding as a component of nutrition programming. Therefore, MSF trained community health workers in the use of the MUAC tape for the purpose of conducting regular active screening and identification of children with SAM within the community. To simplify field operations in a remote, resource-poor setting where malnutrition is highly prevalent, this MSF program used MUAC as both the means for community-based screening and as the case definition of severe wasting that determines admission to therapeutic feeding. All children meeting the admission criteria at community level were referred to the nearest Centre de Santé et de Promotion Sociale [CSPS] for registration, medical examination and initiation of therapeutic feeding. Coherency between the screening tool and the admission criterion minimizes the problem of referral error that often occurs when children are screened by MUAC, but then admitted by WHZ – as these two anthropometric measures do not identify the same group of malnourished children [15]. The fact that children identified in the village were not refused admission at the CSPS encouraged mothers to present with their malnourished children. With a diagnostic tool easy to use and to understand by both mothers and health workers, it might be less difficult to introduce SAM treatment into the standard package of care.

In the Burkina Faso project, MSF piloted the use of a MUAC threshold of ≤118 mm and/or nutritional oedema as the only two admission criteria. The choice of a MUAC admission cut-off at ≤118 mm has been informed by statistical analyses of nutrition surveys conducted by MSF in sub-Saharan Africa that suggest using MUAC >115 mm improves sensitivity for identifying children below WHZ <−3, with minimal decrease in specificity [16]. Although there is no “gold standard” definition for acute malnutrition, field experience has shown that children with a WHZ <−3 exhibit rapid rates of weight gain with therapeutic feeding [17], which suggests benefit from admission to TFP. Thus the MUAC admission criterion was adjusted upwards to increase inclusion of children in the anthropometric category MUAC ≥115 mm but WHZ <−3.

Given the dearth of evidence regarding the use of MUAC as a tool to monitor response to nutritional recovery, the WHO recommends that children admitted by MUAC be discharged according to percent weight gain [18]. However, it would be most consistent to use the same measure for admission and discharge criteria, be it MUAC or WHZ.

Here we report on program data from children admitted to TFP by MUAC, focusing on three main issues regarding the current management of SAM: 1) SAM diagnosis and advantages in using a broader MUAC inclusion criterion versus using combined MUAC and WHZ criteria; 2) the effects of the current WHO discharge criteria on treatment duration; and 3) response of MUAC to treatment and potential to use MUAC as a discharge criteria.

## Methods

### Ethics statement

This study used routine program monitoring data from the MSF nutritional program in Burkina Faso. This program was conducted in collaboration with the Ministry of Health of Burkina Faso via a memorandum of understanding, which is the normal and usual operating procedure for NGOs implementing nutritional programs. No supplementary interventions were given to participants. All data were anonymized when entered into the database and identification numbers were coded. No ethnic or identifying information was included. Individual oral informed consent was obtained from the parent or caregiver at the child's admission to the program.

### Nutrition program overview

In collaboration with the Ministry of Health, in September 2007, MSF launched a TFP in two health districts of the Northern Region in Burkina Faso: Titao (capital of Loroum Province) and Yako (capital of Passore Province). According to the 2006 census, the total populations for Loroum and Passore provinces were 142,853 and 323,222, respectively [19]. In 2009, there were 20 CSPS in Titao and 48 in Yako.

Nutrition indicators for the Northern Region, assessed in June–July 2008, reflected high levels of both stunting and wasting. Stunting (height-for-age <−2 Z WHO) prevalence among children under 5 was 40.8% and prevalence of wasting (WHZ <−2 WHO) was 11.6%, compared to stunting of 38.7% and WHZ <−2 of 12.4% on a national level [20].

As recommended by the national guidelines in Burkina Faso, the nutritional program implemented by MSF was located within the national health system. The program included an inpatient center for complicated cases (Inpatient-Therapeutic Feeding Center or I-TFC, similar to “Stabilization Center” in standard Community-based Therapeutic Care (CTC) terminology) at hospital level, and a series of outpatient units (Ambulatory Therapeutic Feeding Center or A-TFC, similar to “Outpatient Therapeutic Program” in CTC terminology) at CSPS level. As it was not deemed necessary to cover all health structures, some CSPS facilities were selected to deliver A-TFC services according to population density and accessibility. CSPS were identified in such a way that distances remained at an acceptable level and were not considered as a barrier to access for the mothers. The TFP in Yako was composed of 10 A-TFCs (out of the 48 CSPS) and one I-TFC with a capacity of 100 beds located within the district hospital. The TFP in Titao was composed of 12 A-TFCs (out of the 20 CSPS) and one I-TFC with a capacity of 100 beds also at the district hospital.

### Admission and discharge criteria and therapeutic feeding protocol

The MSF program trialed a less restrictive MUAC admission cutoff of ≤118 mm, compared to the current WHO-recommended ≤115 mm. Although weight and height were measured for investigative purposes (height was not systematically recorded for the first 6 months of the program), WHZ tables were not used to determine TFP eligibility. All children aged 6–59 months presenting to one of the 22 CSPS where MSF was present were admitted to the TFP if they had MUAC ≤118 mm and/or oedema. A very small number of special cases (malnourished children below the age of 6 months or above the age of 59 months) were also admitted upon medical decision.

Children were first assessed for bilateral pitting oedema by applying thumb pressure to the dorsum of the foot. All children presenting pitting oedema were eligible for TFP admission regardless of anthropometry, and thus MUAC was not used as an admission criterion if oedema was present. Age was determined either by noting the birth date recorded on the child's vaccination card or by querying the mother. Weight was recorded using a Salter scale to the nearest 100 g, and length was measured in supine position (or standing for children taller than 85 cm) on a Schorr height board to the nearest 1 mm. MUAC was measured with an MSF-designed tape demarcated in 2 mm increments, using even numbers. MUAC, oedema and weight were measured at each visit, and height was measured at admission and at discharge. Children with severe pathology, anorexia, or oedema extending beyond the dorsum of the foot were considered complicated and admitted directly to the I-TFC. After clinical examination and an appetite test, all non-complicated cases were admitted directly into home treatment with weekly follow-up at A-TFC, and referred to the I-TFC only if they developed complications or failed to respond to treatment.

RUTF (Plumpy'nut®, Malaunay, France) (500 kcal/packet) was used for treating all outpatients: 2 packets/day for those weighing <8 kg and 3 packets/day for those ≥8 kg. Therapeutic milk formulations F-75 and F-100 were used for initial nutritional therapy in hospitalized children, while medical complications were addressed. For these children, RUTF was introduced once appetite returned and clinical condition had stabilized. All children also received standard treatment in compliance with WHO recommendations: 5-day course of amoxicillin, and one-time doses of vitamin A, folic acid, and albendazole. Any identified medical conditions were treated concurrently, without fee, according to standard MSF protocols for malaria and other common conditions. Screening for HIV and tuberculosis was offered for all children with clinical suspicion and, starting from 2009, all children admitted in the intensive care unit of the I-TFC were routinely offered HIV testing. All necessary treatment and follow-up consultations were offered free of charge.

Children receiving nutritional intervention were considered as recovered when all of the following criteria were achieved: weight gain >15% of admission weight; minimum length of stay of 4 weeks; and absence of any associated pathology. If admitted with oedema, children were discharged a minimum of 7 consecutive days after its complete resolution. Children were classified as defaulters if they failed to appear for scheduled weekly appointments for 3 consecutive weeks (in A-TFC) or were absent 3 consecutive days (in I-TFC). Non-response was defined as failure to achieve 15% weight gain after 6 weeks in A-TFC followed by good compliance with treatment during supervised feeding in the I-TFC and exclusion of any associated pathology or chronic disease. Children re-presenting to the A-TFC more than two months after being discharged as recovered from the program were recorded as new admissions; the ones presenting within two months after being discharged as recovered were classified as re-admissions.

### Data analysis

Routinely collected data of patients admitted and discharged between September 2007 and March 2009 were systematically entered into a database upon discharge and retrospectively analyzed, including date of admission and exit, demographic information (age, gender, nationality), anthropometric measurements at admission and exit, weekly weight and MUAC, symptoms at admission, main morbidities during follow-up, treatment received, and program outcomes (cured, death, default, non-response, or transfer).

Data were entered daily, using EpiData software, version 3.02 (EpiData Association, Odense, Denmark). Data quality was checked by built-in parameters in EpiData that allowed data entry only within specified range, in addition to systematic clean-up at the end of each month when a list of records with aberrant values was generated and compared to the medical file. If a discrepancy occurred, the value on the medical file was entered.

Data were analyzed using STATA software, version 10 (STATA Corp., College Station, TX, USA). Means of weight-for-height Z score (WHZ), weight-for-age Z score (WAZ), height-for-age Z score (HAZ), and MUAC-for-age Z score (MUAZ), using WHO 2006 growth standards, were calculated using WHO Anthro, version 2.0.4 (WHO, Geneva, Switzerland).

## Results

**Figure 1:**
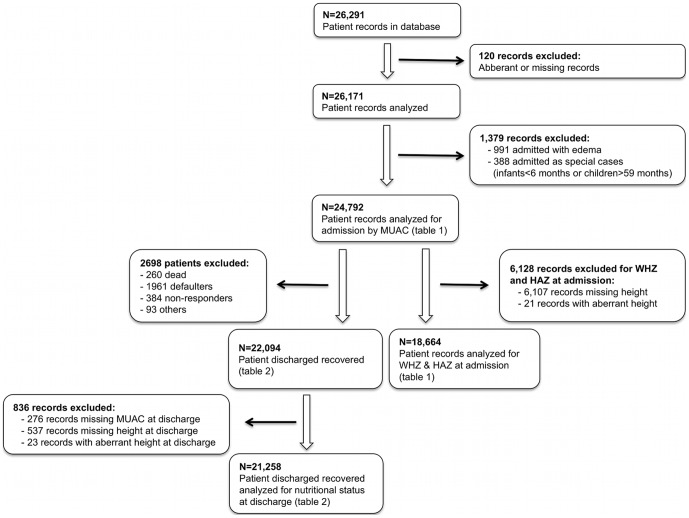
Flowchart describing patient records used in data analysis.

The total number of records in the database from September 2007 to March 2009 was 26,291. In this analysis, 120 records were excluded because of aberrant or missing data (Figure 1).

A total of 26,171 records were analyzed for children admitted to the nutritional program between September 2007 and March 2009. Of all children admitted during this period, 94.7% (24,792) were admitted based on MUAC alone, with the remaining 3.8% (991) admitted based on oedema, and 1.5% (388) admitted as special cases. We focus only on children admitted based on MUAC.

From November 2007 through April 2008, height at admission was not routinely noted. For this reason, only 18,664 out of the 24,792 admissions by MUAC contributed to determination of WHZ and HAZ upon admission.

### Patient admission characteristics

Among children admitted based on MUAC (Table 1), close to half (48.8%) were admitted with MUAC 116–118 mm. Female-to-male ratio of the overall cohort was 1.01, and 88.7% of admissions were of children 6–24 months old. Median age was 13 months in all MUAC admission categories. Within this cohort, a total of 61 children tested positive for HIV and around 80 new cases of TB were treated.

**Table 1:** Patient characteristics at admission by MUAC, Yako and Titao nutritional program, Burkina Faso, September 2007–March 2009.

N (%)	MUAC <100 mm	MUAC 100–110 mm	MUAC 112–114 mm	MUAC 116–118 mm	Total
***Number of children admitted***	***1,306 (5.3)***	***6,018 (24.3)***	***5,378 (21.7)***	***12,090 (48.8)***	***24,792 (100.0)***
**Sex**					
Female	666 (51.0)	3,112 (51.7)	2,745 (51.0)	5,950 (49.2)	12,473 (50.3)
Male	640 (49.0)	2,906 (48.3)	2,633 (49.0)	6,140 (50.8)	12,319 (49.7)
**Age (months)**					
6–12	635 (48.6)	2,871 (47.7)	2,468 (45.9)	5,379 (44.5)	11,353 (45.8)
13–24	533 (40.8)	2504 (41.6)	2,282 (42.4)	5,311 (43.9)	10,630 (42.9)
25–36	120 (9.2)	568 (9.4)	554 (10.3)	1,217 (10.1)	2,459 (9.9)
37–59	18 (1.4)	75 (1.2)	74 (1.4)	183 (1.5)	350 (1.4)
Median [IQR]	13 [9–19]	13 [10–19]	13 [10–20]	13 [10–21]	13 [10–20]
**Place of admission**					
A-TFC	856 (65.5)	5,405 (89.8)	5,040 (93.7)	11,498 (95.1)	22,799 (92.0)
I-TFC	450 (34.5)	613 (10.2)	338 (6.3)	592 (4.9)	1,993 (8.0)
**Nutritional status at admission**					
MUAC, mean ± SD	93.0±5.3	107.0±3.2	113.0±1.0	117.0±1.0	112.5±6.6
WHO MUAC/AGEZ, mean ± SD	−5.3±0.6	−3.9±0.5	−3.2±0.4	−2.8±0.4	−3.3±0.8
WHO WAZ, mean ± SD	−5.5±0.8	−4.3±0.9	−3.7±0.8	−3.4±0.8	−3.8±1.0
***Number of records excluding missing or aberrant height data (N)***	***854***	***4,253***	***3,993***	***9,654***	***18,664***
WHO WHZ, mean ± SD	−5.0±1.1	−3.9±0.9	−3.4±0.8	−3.1±0.8	−3.4±1.0
Admissions with <−3 WHZ (%)	829 (97.1)	3,692 (86.8)	2,783 (69.7)	5,041 (52.7)	12,345 (66.1)
WHO HAZ, mean ± SD	−3.9±1.5	−2.9±1.5	−2.5±1.4	−2.3±1.4	−2.5±1.5

Of the 18,664 children for whom height data was available, a total of 66.1% (9,100) had a WHZ <−3 SD (WHO standard). Of those admitted with MUAC 116–118 mm, mean WHZ was −3.1±0.8 SD and 52.7% (5,041) had a WHZ <−3 SD. 76% of the cohort were admissible by either MUAC <115 mm or WHZ <−3 criteria, meaning that almost 1 in 4 children admitted did not meet either currently WHO-accepted anthropometric definition of SAM (Table 1).

### Outcomes and treatment response

Of children admitted to TFP by MUAC, 8% were hospitalized on day of admission in the I-TFC for management of a significant pathology. An additional 10.1% were transferred to I-TFC on a subsequent visit for either illness of poor weight gain, resulting in a total of 81.9% of children who were treated exclusively as out patients (Table 2).

**Table 2:** Patient outcomes of children admitted by MUAC, Yako and Titao nutritional program, Burkina Faso, September 2007–March 2009.

N (%)	MUAC <100 mm	MUAC 100–110 mm	MUAC 112–114 mm	MUAC 116–118 mm	Total
***Number of children discharged***	***1,306 (5.3)***	***6,018 (24.3)***	***5,378 (21.7)***	***12,090 (48.7)***	***24,792 (100.0)***
**Outcome**					
Cured	1,105 (84.6)	5,301 (88.1)	4,813 (89.5)	10,875 (90.0)	22,094 (89.1)
Dead	52 (4.0)	73 (1.2)	48 (0.9)	87 (0.7)	260 (1.0)
Defaulter	134 (10.3)	553 (9.2)	432 (8.0)	842 (7.0)	1,961 (7.9)
Non-responder	14 (1.1)	64 (1.1)	71 (1.3)	235 (1.9)	384 (1.5)
Others	1 (0.1)	27 (0.4)	14 (0.3)	51 (0.4)	93 (0.4)
**Type of treatment**					
A-TFC only	734 (56.2)	4,823 (80.1)	4,579 (85.1)	10,180 (84.2)	20,316 (81.9)
I-TFC ± A-TFC	572 (43.8)	1,195 (19.9)	799 (14.9)	1.910 (15.8)	4,476 (18.1)
***Number of records excluding missing or aberrant MUAC or height at discharge***	***1,042***	***5,083***	***4,661***	***10,472***	***21,258***
**Nutritional status at discharge (for children discharged as cured)**					
MUAC, mean ± SD	119.4±8.0	126.8±7.0	131.0±6.4	133.8±6.5	130.0±8.0
WHO MUAC/AGEZ, mean ± SD	−2.6±0.8	−1.8±0.7	−1.4±0.7	−1.2±0.6	−1.5±0.6
WHO WHZ, mean ± SD	−2.3±1.1	−1.8±1.0	−1.5±0.9	−1.3±0.9	−1.5±0.9
WHO WAZ, mean ± SD	−3.7±1.0	−2.8±1.0	−2.4±0.9	−2.1±0.9	−2.4±1.0
WHO HAZ, mean ± SD	−3.8±1.6	−2.9±1.5	−2.5±1.4	−2.2±1.4	−2.5±1.5

Overall, 89.1% of patients admitted by MUAC were discharged as recovered, while 7.9% defaulted, 1.5% failed to respond to treatment, and 1.0% died (Table 2). Children with MUAC <100 mm at admission (i.e. more severely malnourished; mean WHZ ± SD −5.0±1.1) were more likely to be hospitalized and experienced the poorest outcomes compared with the other (higher) MUAC ranges; these children had the lowest rate of recovery (84.6%) and highest death (4.0%) and defaulter (10.3%) rates. Non-response rate was consistent (1.1–1.3%) for the MUAC groups of children <115 mm but was higher (1.9%) for those admitted with MUAC 116–118 mm (*P*<0.001) (Table 2).

Anthropometric measurements for those discharged as recovered showed that all the children admitted by MUAC responded well to treatment and were discharged from the program with improved anthropometric measurements (Table 2). Among children who recovered, substantial gains in MUAC and WHZ were seen. Mean MUAC at discharge was ≥125 mm in all MUAC categories except those admitted with MUAC <100 mm, and gain of at least 1.5 Z-score in MUAC-for-age in all categories was recorded. For those children whose height measurements were available, gain of 1.5–2 Z-score in WHZ in all MUAC categories was observed, achieving an average WHZ >−2 at discharge (again, except those admitted with MUAC <100 mm who achieved an average WHZ of −2.3). Height-for-age did not change significantly during treatment.

Average weight gain over the entire treatment was 5.4 g/kg/day across all categories and was inversely related to MUAC upon admission (Table 3). Average length of stay in the program to reach discharge (>15% weight gain) was 54 days, and was greater for the less severely malnourished (MUAC 116–118 mm) compared to those whose MUAC was ≤114 mm upon admission (*P*<0.001).

**Table 3:** Treatment response of recovered children admitted by MUAC, with breakdown by MUAC category, Yako and Titao nutritional program, Burkina Faso, September 2007–March 2009.

	MUAC <100 mm (n = 1,105)	MUAC 100–110 mm (n = 5,301)	MUAC 112–114 mm (n = 4,813)	MUAC 116–118 mm (n = 10,875)	Total (n = 22,094)	*P* value for trend
Average weight gain, g/kg/day	8.0±4.3	6.1±7.3	5.3±2.9	4.7±4.9	5.4±5.3	<0.001
Average length of stay, days	46.6±22.0	50.6±24.5	52.9±25.9	56.6±28.0	53.9±27.7	<0.001
Average MUAC gain, mm/day	0.66±0.31	0.48±0.27	0.42±0.25	0.37±0.23	0.42±0.26	<0.001

Among children admitted with MUAC, those with a MUAC ≤114 mm or a WHZ <−3 at admission, corresponding to the children that would fulfill the current WHO admission criteria, had a significantly higher average weight gain (5.7 vs 4.5 g/kg/day; *P*<0.001) and a shorter length of stay (52 vs 59 days; *P*<0.001) than the ones admitted with a WHZ ≥−3 (Table 4).

**Table 4:** Treatment response of recovered children admitted by MUAC, with comparison of children fulfilling the current WHO criteria and those who didn't, Yako and Titao nutritional program, Burkina Faso, September 2007–March 2009.

	MUAC ≤114 mm or WHZ <−3 (n = 15273)	MUAC 116–118 mm & WHZ ≥−3 (n = 5985)	Total (n = 21258)	*P* value
Average weight gain, g/kg/day	5.7±4.9	4.5±6.0	5.4±5.3	<0.001
Average length of stay, days	52.0±25.5	58.7±29.0	53.9±27.7	<0.001
Average MUAC gain, mm/day	0.44±0.26	0.37±0.23	0.42±0.26	<0.001

**Figure 2:**
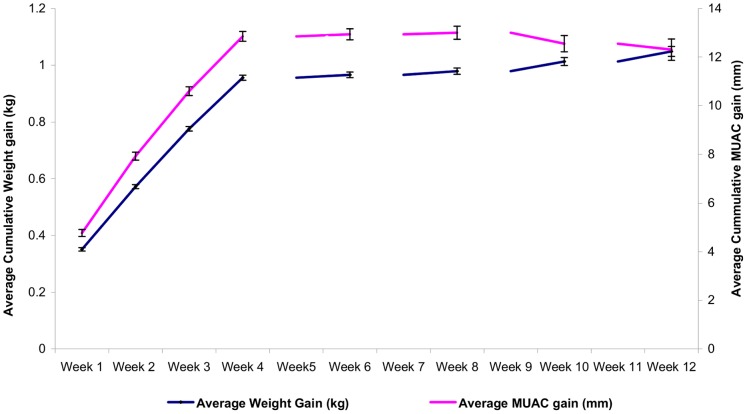
Average cumulative weight gain with average cumulative MUAC gain, MSF Therapeutic Feeding Program, Burkina Faso. Rate of average weekly MUAC gain mirrors average weekly weight gain for all 22,094 children who met discharge criteria for recovery.

MUAC gain over the entire course of treatment was 0.42 mm/day on average and parallelled weight gain (Figure 2). The same trend for average weekly weight gain according to level of malnutrition was noted for MUAC gain.

## Discussion

### Simplicity and feasibility of MUAC-only admission

To our knowledge this is the first report of a large-scale MUAC-based TFP, with outcomes reported according to MUAC upon admission. The demographic profile of the children admitted to this TFP is similar to that reported elsewhere for Sahelian populations where wasting is the predominant form of SAM and stunting is highly prevalent [17]. Although TFPs usually target children 6–59 months old, admissions typically cluster around the 12–23 month age group, since wasting often peaks in this age group [21]. This was the case in Burkina Faso in its 2009 National Nutrition Survey [20]. The median age of 13 months in this cohort also reflects an age bias inherent to the application of a uniform MUAC cut-off applied across the 6–59 month age range. MUAC thus has the advantage of being more sensitive for younger age groups, and including more children during the critical development window of the first two years of life. Younger children, at higher mortality risk because of age, are included earlier in the wasting process before the onset of medical complications.

Interestingly, the sex ratio was close to 1 in this program, whereas other TFPs that use the WHO gender specific WHZ tables report a predominance of male admissions [17]. The gender discrepancy that results from using WHZ tables is due to lower weights applied for girls, which makes it less likely to be identified as malnourished. In this program, MUAC does not appear to result in a gender bias and may prove advantageous as it is unlikely that male children are disproportionately affected by malnutrition.

Upward adjustment of the MUAC admission threshold for this nutrition program demonstrates a gain in sensitivity in that a substantial number of children with WHZ <−3, but MUAC equal to 116–118 mm, were included. The programmatic “price” for this increase in sensitivity was an additional caseload of 4613 patients who met neither WHO-accepted admission criterion. Although this increased workload does pose concern in terms of resources required to run the program, notably RUTF, potentially there are gains in terms of efficient use of health workers' time as well as the benefits offered by early identification of malnourished children and avoidance of hospitalization. Furthermore, those children with MUAC 116–118 mm and WHZ >−3 demonstrated rates of weight gain typically seen in TFPs, suggesting real benefit from treatment.

### Treatment response

Treatment outcomes even in children with lowest MUAC upon admission in this cohort (<100 mm) exceeded SPHERE 2011 performance standards [22] for recovery, defaulting, and mortality, and improved substantially for the other three MUAC groups (100–110 mm; 112–114 mm and 116–118 mm). Average weight gain of 5.4 g/kg/d for the children in this cohort compared favorably to that reported by other community-based nutritional programs, where average weight gain for children recovering from acute malnutrition varies between 3 to 6.8 g/kg/d [23]. The children with WHZ >−3 experienced slightly lower average daily weight gain (4.5 vs 5.7 g/kg/d) compared to those with WHZ <−3, but still substantially better than the ones reported for moderately malnourished children treated with RUTF over a similar time period [24], [25]. The treatment response noted in this sub-group of the cohort advocates therefore in favor of treating them as severely malnourished.

Our findings appear also to be the first to document a similar profile in rate of MUAC gain according to initial severity of malnutrition. Whereas previous interventions have reported a mean MUAC gain of 0.3 mm/day (sometimes based on treatment with higher amounts of RUTF) [14], we observed MUAC gains of 0.4–0.6 mm/day, with rate of daily MUAC gain directly related to rate of daily weight gain (Table 3). These rates of gain translate to absolute MUAC gains of approximately 15 mm per month. MUAC gain could therefore be useful for setting alternative goals for nutritional recovery, in other words application of a MUAC threshold for discharge in lieu of a weight gain criterion.

### Discharge criteria reconsidered

A major challenge in the Burkina Faso program was the use of WHO-recommended 15% weight gain as the discharge criterion. Paradoxically, using >15% weight gain to determine nutritional recovery and program discharge resulted in longer treatment for less malnourished children, as measured by average length of stay in the program, and shorter treatment for more severely malnourished children (Table 3). The more malnourished children (eg. lower MUAC) met the discharge criterion more rapidly and thus spent less time benefitting from nutritional rehabilitation. Conversely, the non-response rate is significantly higher among the least malnourished children, compared to those with MUAC <116 mm. This appears to be related to timing of response to treatment: virtually all of the rapid weight gain is seen in the first 4–5 weeks in therapeutic feeding (Figure 2). If the child has not gained 15% of admission weight within this window, the likelihood of meeting this criterion significantly diminishes and suggests little added benefit to prolonged courses of treatment.

Since average weekly MUAC gain and average weekly weight gain tracked closely together (Figure 2), MUAC gain may be useful as a program monitoring and discharge tool. Using the same measurement tool for screening, admission, and discharge (as well as possibly for treatment monitoring and follow-up) could make programs more coherent and understandable to caregivers. Using MUAC as both admission and discharge criteria could also simplify nutrition programming and help direct more resources to those children more severely malnourished, by keeping those with lower MUAC under care until they achieve a MUAC that is associated with lowered mortality risk, generally estimated to be >125 mm [12]. In the MSF program in Burkina Faso, since April 2009 children are admitted with MUAC <120 mm, and discharged once the MUAC is >124 mm with a minimum duration of 4 weeks of therapeutic feeding. This change has improved program coherency: children who enter the program at lowest MUAC have the longest lengths of stay, while those who are admitted close to the threshold achieve discharge criteria within 4 weeks (unpublished data). Analysis of program outcomes using a MUAC discharge criterion will be addressed in a subsequent publication.

### Study limitations

The results reported here must be interpreted in light of several limiting factors. These analyses were performed on program data and cannot therefore be attributed to the entire population of young children in northern Burkina Faso; the findings apply only to those children who were brought by their caregivers to the screening sessions or to the health centers.

Although TFP staff were trained and continuously supervised, the quality of measurements is reflective of what one might find in any given large TFP. Because of the MSF MUAC tape design (demarcated in 2 mm increments and using only even numbers), only even numbers were recorded on the patient chart and the maximum eligible admission value was designated as 118 mm. A number of children measuring MUAC = 118 mm would therefore likely have a measurement of 117 mm or 119 mm, if a measuring tape demarcated in 1 mm increments was used. However, given the large cohort size, measurement errors may cancel each other out. For the first 6 months of this program, height was not consistently measured upon admission; during this time the relative proportion of more severely wasted children was higher. Therefore, the reported results in terms of WHZ for the most severely wasted children under-represent this category. Almost all of the children in this cohort are under 2 years of age, and are in a physiologic period of rapid growth, regardless of nutritional status. Rates of MUAC gain may be slower in older children.

### Conclusion

Large-scale, community-based treatment of severely malnourished children using an adapted MUAC threshold may be able to replace the more complicated anthropometric index of WHZ. Based on simplicity and predictive value of mortality, MUAC-based detection using a fixed cutoff represents an opportunity to easily screen large numbers of children and admit them efficiently to a TFP using the same anthropometric measurement. MUAC-based programs will preferentially identify younger children as malnourished, which is a beneficial bias since children of this group are more vulnerable to illness and at higher risk of death. A more generous inclusion criterion may thus identify malnourished children earlier, when they can be treated easily and effectively, but remain sufficiently specific as evidenced by rapid weight gain. The current WHO recommendation of 15% weight gain as a threshold for discharge is paradoxically disadvantageous for the most severely malnourished children, according to our findings. Beyond use as sole admission criterion, MUAC shows potential as a tool for monitoring response to nutritional rehabilitation as well as a discharge criterion.
